# Analyses of murine GBP homology clusters based on *in silico*, *in vitro *and *in vivo *studies

**DOI:** 10.1186/1471-2164-9-158

**Published:** 2008-04-10

**Authors:** Alexandra Kresse, Carolin Konermann, Daniel Degrandi, Cornelia Beuter-Gunia, Jan Wuerthner, Klaus Pfeffer, Sandra Beer

**Affiliations:** 1 Institute of Medical Microbiology and Hospital Hygiene, Heinrich-Heine-University, Universitaetsstrasse 1, 40225 Duesseldorf, Germany

## Abstract

The interactions between pathogens and hosts lead to a massive upregulation of antimicrobial host effector molecules. Among these, the 65 kDa guanylate binding proteins (GBPs) are interesting candidates as intricate components of the host effector molecule repertoire. Members of the GBP family are highly conserved in vertebrates. Previous reports indicate an antiviral activity of human GBP1 (hGBP1) and murine GBP2 (mGBP2). We recently demonstrated that distinct murine GBP (mGBP) family members are highly upregulated upon *Toxoplasma gondii *infection and localize around the intracellular protozoa *T. gondii*. Moreover, we characterised five new mGBP family members within the murine 65 kDa GBP family. Here, we identified a new mGBP locus named *mGbp11*. Based on bacterial artificial chromosome (BAC), expressed sequence tag (EST), and RT-PCR analyses this study provides a detailed insight into the genomic localization and organization of the mGBPs. These analyses revealed a 166-kb spanning region on chromosome 3 harboring five transcribed mGBPs (*mGbp1, mGbp2, mGbp3, mGbp5*, and *mGbp7*) and one pseudogene (pseudo*mGbp1*), as well as a 332-kb spanning region on chromosome 5 consisting of six transcribed mGBPs (*mGbp4, mGbp6, mGbp8, mGbp9, mGbp10*, and *mGbp11*), and one pseudogene (pseudo*mgbp2*). Besides the strikingly high homology of 65% to 98% within the coding sequences, the mGBPs on chromosome 5 cluster also exhibit a highly homologous exon-intron structure whereas the mGBP on chromosome 3 reveals a more divergent exon-intron structure. This study details the comprehensive genomic organization of mGBPs and suggests that a continuously changing microbial environment has exerted evolutionary pressure on this gene family leading to multiple gene amplifications. A list of links for this article can be found in the Availability and requirements section.

## Background

The guanylate binding proteins (GBPs) were first described in 1979 when Gupta et al. identified a 67 kDa protein induced in human fibroblasts after interferon γ (IFNγ) stimulation [1]. Some years later, it was shown that two orthologous proteins are expressed in murine fibroblasts after stimulation with IFNγ [2]. Besides the strong inducer IFNγ, the GBPs can also be induced by type I interferons [2-6], tumor-necrosis-factor α (TNF-α), interleukin-1β (IL-1β), IL-1α [7,8], and TLR agonists [5].

Human and murine GBPs possess the unique ability to bind to agarose-immobilized GMP (guanosine monophosphate), GDP (guanosine diphosphate), and GTP (guanosine triphosphate) with the same affinity, thereby differing from heterotrimeric or Ras-like GTP-binding proteins [2]. In addition, they hydrolyse GTP not only to GDP but also to GMP [9]. Further biochemical properties of the GBPs are the low binding affinity to nucleotides, their stability in the absence of guanine nucleotides and their high turnover GTPase activity [10]. Remarkably, the sequence of the common G4-motif N/TKxD is modified in the GBPs to the unique T(L/V)RD motif [10]. In the case of hGBP1 a nucleotide-dependent oligomerization and concentration-dependent GTPase activity has been observed [11]. These biochemical properties classified the GBPs as distantly related family members of the dynamin superfamily despite the lack of any sequence homology of the primary sequences [11,12]. The similarity to the dynamin family is further corroborated by the analysis of the crystal structure of hGBP1. It has an amino-terminal globular domain containing the GTP binding region and an elongated carboxy-terminal series of α-helices. The GBPs possess the common 'dynamin domain structure' with a GTPase domain (~300 residues), a 'middle' or 'assembly' domain (150–200 residues) and a GTPase effector domain (~100 residues) [11].

Although, the GBPs have been discovered almost 30 years ago, only little is known about their biological function. It has been suggested that the GBPs are important for cell growth regulation as demonstrated for mGBP2 and hGBP1 [7,13]. Both, hGBP1 and mGBP2 also alter matrix metalloproteinase (MMP) gene expression and thereby change cellular interactions with the extracellular environment [14]. In addition, hGBP1 was reported to be involved with paclitaxel resistance in ovarian cancer cell lines [15]. Further studies revealed that hGBP1 and mGBP2 exhibit a moderate antiviral activity against vesicular stomatitis virus (VSV) and encephalomyocarditis virus (EMCV) [16,17]. Recently, we have demonstrated that mGBPs are highly upregulated in mice after infection with *Listeria monocytogenes *or *Toxoplasma gondii*, and localize around the parasitophorous vacuole of *T. gondii*, thus suggesting that the mGBPs play a role in the defense against intracellular bacteria and apicomplexa [5].

Besides humans and mice the GBPs have been found in rats [18], chicken [19], fish [20], and several other vertebrate species. In humans seven orthologs and at least one pseudogene have been identified [21,22]. In mice five GBPs have been described [2,23,24]. Recently, one additional mGBP was discovered by an *in silico *study [21]. In search of new IFNγ regulated genes using Affymetrix analyses we independently identified *mGbp6*, *mGbp7*, and *mGbp8 *[5]. Further comprehensive genome and sequence analyses yielded two more homologous genes, named *mGbp9 *and *mGbp10 *[5]. The subsequent investigation of mGBP gene loci revealed an incorrect assembly concerning the *mGbp8 *locus in the genome databases (Ensembl, NCBI). Thus, to clarify the genomic organization of mGBP coding genes, we used BAC and EST sequences obtained from NCBI. Based on these sequence analyses we were able to identify another mGBP locus named *mGbp11*. In this study, we address the genomic organization and localization of each mGBP family member and present a revised assembly of the murine GBP homology clusters on chromosomes 3 and 5.

## Results

### The mGBP genes are arranged in two clusters located on chromosomes 3 and 5

**Figure 1:**
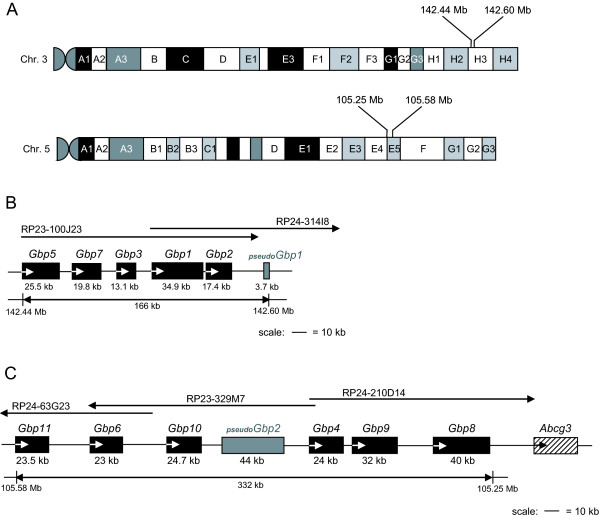
**mGBP genes cluster on chromosomes 3 and 5**. **A. **One mGBP cluster is located within the H3 region on chromosome 3 (142.44 MB to 142.60 MB) and the other within the E5 region on chromosome 5 (105.25 MB to 105.58 MB). Genomic organization of the mGBP genes clustered on chromosome 3 **(B) **and chromosome 5 **(C). **Each gene locus is illustrated as a black rectangle, pseudogenes are shown in grey. The locus of the flanking gene *Abcg3 *is shaded. The approximate length of each gene is depicted below the corresponding rectangle. Transcriptional direction is indicated by white arrows. The approximate length of each cluster is shown at the bottom of the respective figure. The BACs used in this study are stated above the corresponding region of the chromosomes. The scale bar equals a length of 10 kb.

Although the first guanylate binding protein had been described almost 30 years ago further family members have been discovered lately. A recent *in silico *study has proposed a number of six mGBPs and three pseudogenes [21]. We recently raised the number up to ten mGBP members [5]. In this study, we performed extensive homology searches on the whole genome to identify further mGBP loci. Single exons of known mGBPs were used for homology searches against murine genome databases using the BlastN algorithm [25]. Thereby, another mGBP family member was discovered, now designated mGBP11 (Acc. No. EU304258). The *mGbp11 *locus was confirmed by BAC analyses. Hence, we extend the family to eleven mGBPs and two pseudogenes which all cluster on two chromosomes. One gene cluster is located on chromosome 3 within the H3 region and the second gene cluster is found within the E5 region on chromosome 5 (Fig. 1A). For further analysis of these two gene clusters we used BAC sequences obtained from NCBI. On chromosome 3 the BACs RP23-100J23 and RP24-314I8 cover the respective chromosomal regions between 142.44 MB and 142.60 MB harboring *mGbp1, mGbp2, mGbp3, mGbp5, mGbp7*, and a pseudogene named pseudo*mGbp1 *(Fig. 1B). This gene cluster spans approximately 166 kb. The length of each gene locus ranges from 3.7 kb (pseudo*mGbp1*) to 34.9 kb (*mGbp1*). The mGBPs located on chromosome 3 are all transcribed from the positive strand.

Further, we used the BACs RP24-63G23, RP23-329M7 and RP24-210D14 for analyses of the region between 105.25 MB and 105.58 MB on chromosome 5 (Fig. 1C). By means of these BAC sequences we were able to determine the precise loci for *mGbp4*, *mGbp6 *(formerly *mpa2l*), *mGbp8*, *mGbp9*, *mGbp10*, *mGbp11*, and the pseudogene pseudo*mGbp2*. This cluster has approximately twice the size of the cluster on chromosome 3 with an extension of 332 kb. The length of each gene locus on chromosome 5 ranges from 23 kb (*mGbp6*) to 44 kb (pseudo*mGbp2*). In contrast to the mGBPs located on chromosome 3 the mGBPs on chromosome 5 are all transcribed from the negative strand.

### Conserved exon-intron structure in the mGBPs

**Figure 2:**
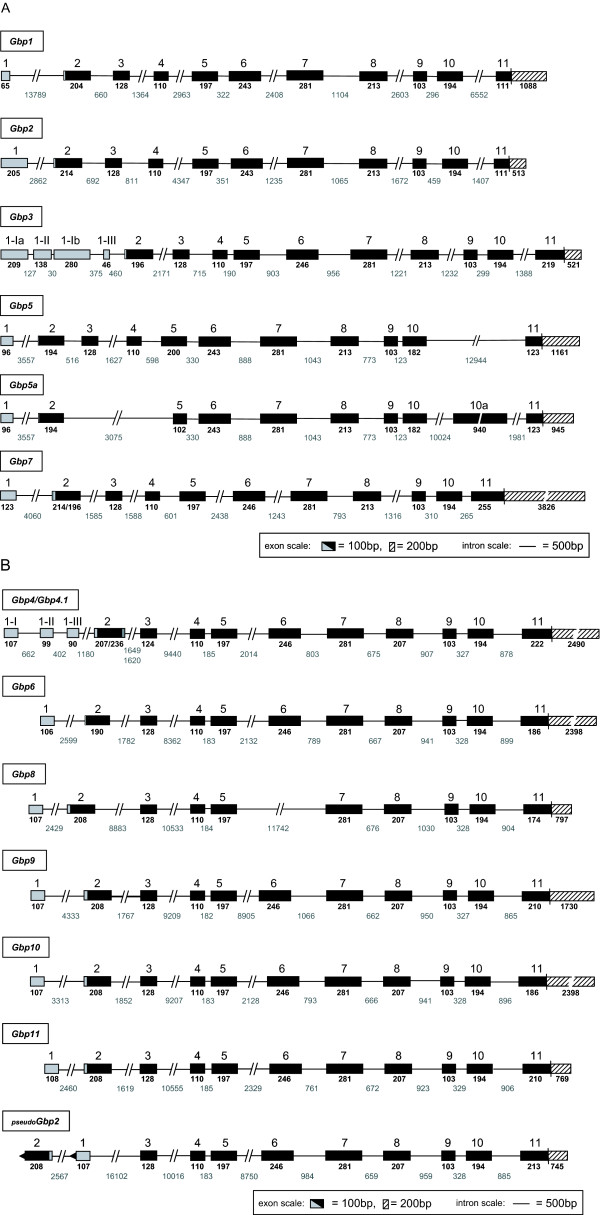
**Genomic organization of mGBP genes on chromosome 3 (A) and chromosome 5 (B)**. Translated exons are illustrated in black, 5' untranslated exons in grey and 3' untranslated exons are shaded. The alternative 5' non-coding exons of mGBP3 (I-a, II, I-b, and III) and mGBP4 (I, II, and III) are numbered separately. The mRNAs for mGBP5 and mGBP5a are differentially spliced transcripts encoded by the same genomic locus. However, since alternative splice acceptor sites in exon 5 are used and an alternative exon 10a is inserted in the different mRNAs two schematic illustrations are provided. The inversion of the first two exons of pseudogene pseudo*mGbp2 *is indicated by black arrowheads. The length (bp) of each exon and intron is specified. The depicted scale bars correspond to the different rulers used for introns, coding or non-coding exons.

Besides their clustering on two chromosomes, the mGBPs share a highly similar genomic structure (Fig. 2). All members of the 65 kDa mGBP gene family consist of eleven exons, except *mGbp8 *which lacks exon 6. Since exon 6 is composed of 246 basepairs no frameshift is generated. The cloning and sequencing of mGBP8 cDNA confirmed these genomic findings. Interestingly, the translation of all mGBPs starts within exon 2.

The size of the first non-coding exon of the mGBPs on chromosome 3 ranges from 65 bp (mGBP1) to 205 bp (mGBP2) (Fig. 2A). For mGBP3 several alternative 5' UTR exons were identified. In the database 26 EST and cDNA sequences which cover the 5' UTR from mGBP3 were found: seven ESTs contain exon 1-Ia upstream of exon 2, three sequences harbor exon 1-Ia and 1-Ib in combination, 16 sequences comprise exon 1-II and only one EST was found having exon 1-III as a 5' non coding exon. For mGBP7 two different splice sites in the 5' UTR of exon 2 were observed as reported previously [21]. In every mGBP locus the exons 3 (128 bp), exons 4 (110 bp), exons 5 (197 bp), exons 7 (281 bp), exons 8 (213 bp), exons 9 (103 bp), and exons 10 (194 bp) cover the same size, except mGBP5 which has an exon 5 with 200 bp instead of 197 bp and an exon 10 with 182 bp instead of 194 bp. Moreover, for mGBP5 an alternative splice form, mGBP5a, which lacks exons 3, exon 4, a part of exon 5, and possesses an exon 10a, has been described [24]. Exons 6 of mGBP1, mGBP2, and mGBP5 consist of 243 bp whereas exons 6 of the other mGBPs span 246 bp.

Interestingly, the mGBPs on chromosome 5 show even higher similarities concerning the genomic organisation (Fig. 2B). The first exons of these mGBPs have nearly identical sizes (between 106 and 108 bp). Only for mGBP4 alternative 5' non-coding exons were observed (1-I, 1-II, and 1-III). EST sequences harboring either exon 1-I or exon 1-II as well as all three alternative 5' UTR exons were found in the database. Furthermore, we were able to identify an alternative splice form of mGBP4. Due to a mutation at the splice donor site in intron 2 two different transcripts, named mGBP4 and mGBP4.1, are generated [26]. Except exon 3 of mGBP4, all mGBPs on chromosome 5 share identical sizes of the individual coding exons 3 to 10. Even the intron sequences are highly conserved in these genes. In particular, intron 4, intron 7, intron 8, intron 9, and intron 10 exhibit high similarities. Comparable to all other mGBPs the pseudogene pseudo*mGbp2 *shows a highly conserved exon-intron structure. Yet, an inversion of 'exon 1' and 'exon 2' results in a non-functional locus. Taken together, due to their high structural homologies these loci seem to be duplicated quite recently.

### In vitro and in vivo analyses of the new mGBP members

After determination of the precise exon-intron structure of each mGBP we confirmed the predicted sequences by cloning and sequencing the corresponding cDNAs out of IFNγ stimulated macrophages. These studies revealed that mGBP11 has a premature stop codon within exon 8 leading to an ORF sequence with only 1329 bp. The amino acid sequence and the GTP-binding motif are depicted in additional file 1.

**Figure 3:**
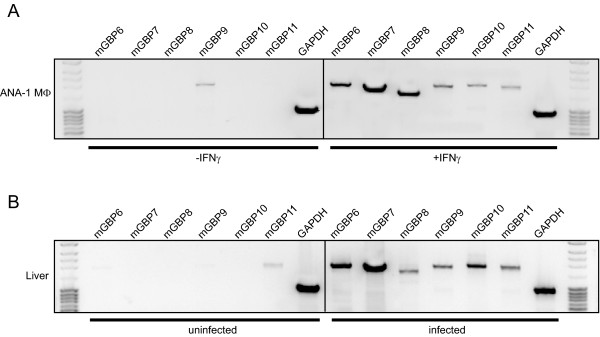
**Transcriptional analyses of mGBP6, mGBP7, mGBP8, mGBP9, mGBP10, and mGBP11**. Amplification of mGBP6, mGBP7, mGBP8, mGBP9, mGBP10, and mGBP11 using specific primers listed in additional file 3 and cDNAs derived from RNA of unstimulated and IFNγ stimulated ANA-1 macrophages **(A)**, and RNA from liver of uninfected and *L. monocytogenes *infected C57BL/6 mice **(B)**. GAPDH primers were used as an internal control.

In order to elucidate the basal expression and inducibility of the new mGBP family members RT-PCR analyses were performed. Therefore, we compared the expression levels in unstimulated and IFNγ stimulated ANA-1 macrophages (Fig. 3A). The mGBPs were only detectable upon stimulation, except mGBP9, which was already expressed in unstimulated cells. Besides IFNγ stimulation, the infection of C57BL/6 mice with *L. monocytogenes *led to a rapid upregulation of all tested mGBPs in the liver (Fig. 3B). Interestingly, slight mGBP11 RNA expression was detectable in the liver of uninfected mice.

### Sequence alignments and homologies of the mGBPs

**Figure 4:**
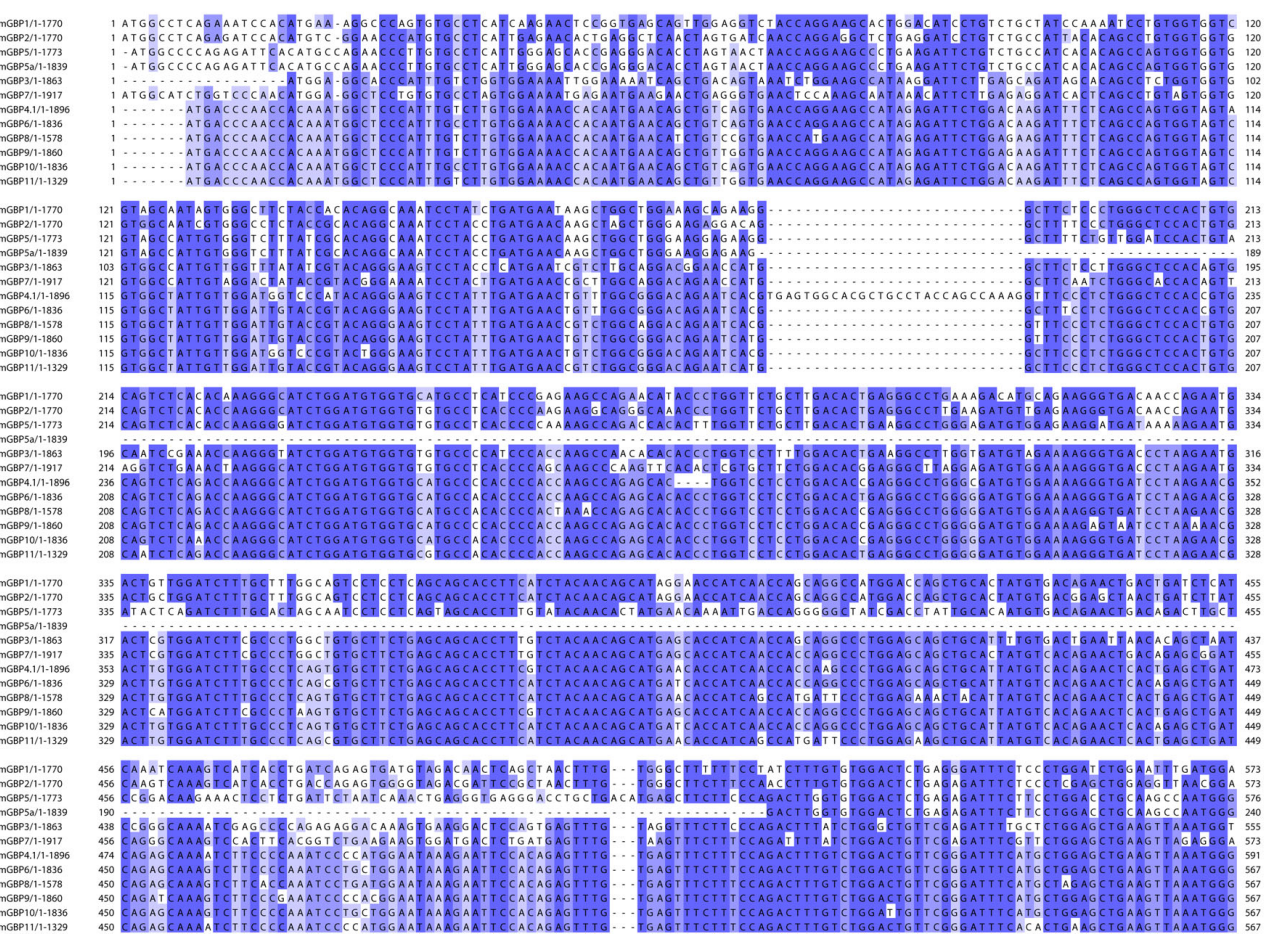
**ClustalW alignment of ORF sequences of the eleven 65 kDa mGBP members**. The alignment-layout was edited with the JalView software. Basepairs identical in all eleven mGBPs are marked in dark blue, identical in ≥75% in medium blue and identical in ≥50% in light blue. Red boxes highlight the conserved GTP binding motifs: P-loop (G1), Switch I (G2), Switch II (G3) and G4. This figure shows the upper quartile, for the full image please see additional file 2.

Based on ORF sequences of the 11 mGBPs we accomplished a cDNA alignment using the ClustalW algorithm (Fig. 4 and additional file 2). As shown in the alignment, the mGBPs share a high degree of homology throughout their coding sequences whereas the 5' part is the most conserved region of the mGBPs. Especially, mGBP6 and mGBP10 are the most homologous mGBPs differing in only 30 basepairs within an ORF sequence of 1836 bp. The conserved 5' part codes for the four typical GTP binding motifs. Although there are some differences in the nucleotide sequence of the GTP binding motifs the amino acid sequences are almost identical (Table 1). In detail, the G1 motifs of mGBP2, mGBP3, mGBP5/5a, mGBP6, mGBP7, mGBP8, mGBP9, and mGBP11 are identical, only the G1 motifs of mGBP1 and mGBP10 differ in two amino acids, and the G1 motif of mGBP4/4.1 differ in three amino acids. The G2 and G3 motifs are the same among all mGBPs. Regarding the G4 domain the mGBPs can be divided into the two subgroups 'TVRD' (mGBP4.1, mGBP6, mGBP7, mGBP8, mGBP9, and mGBP11) and 'TLRD' (mGBP1, mGBP2, and mGBP5), except mGBP3 with a 'AVRD' and mGBP10 with a 'IVRD' motif (Table 1). Most likely the functional consequences are minor since the amino acid variations in different G4 motifs lead to conservative exchange of aliphatic (L/V) and hydrophobic (T/A/I) amino acids. Interestingly, the mGBPs from the 'TLRD' group possess a C-terminal CaaX motif, which can be modified by isoprenylation [27]. Overall, the 3' parts of the mGBPs are more divergent than the 5' parts.

**Table 1:** Comparison of the conserved G1, G2, G3, and G4 motifs of the mGBPs. The canonical GTP motifs of all mGBPs and the overall consensus sequence are shown. Amino acid substitutions are highlighted in bold.

	**G1**	**G2**	**G3**	**G4**
**mGBP1**	G**F**Y**H**TGKS	T	DTEG	T**L**RD
**mGBP2**	GLYRTGKS	T	DTEG	T**L**RD
**mGBP3**	GLYRTGKS	T	DTEG	**A**VRD
**mGBP4**	G**WSH**TGKS	-	----	----
**mGBP4.1**	G**WSH**TGKS	-	DTEG	TVRD
**mGBP5**	GLYRTGKS	T	DTEG	T**L**RD
**mGBP5.a**	GLYRTGKS	-	----	T**L**RD
**mGBP6**	GLYRTGKS	T	DTEG	TVRD
**mGBP7**	GLYRTGKS	T	DTEG	TVRD
**mGBP8**	GLYRTGKS	T	DTEG	TVRD
**mGBP9**	GLYRTGKS	T	DTEG	TVRD
**mGBP10**	G**WS**RTGKS	T	DTEG	**I**VRD
**mGBP11**	GLYRTGKS	T	DTEG	TVRD

**consensus**	**GxxxTGKS**	**T**	**DTEG**	**x(V/L)RD**

**Figure 5:**
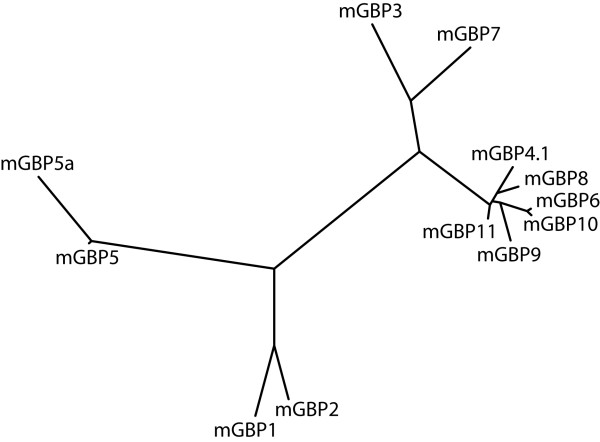
**Phylogenetic tree of the mGBP cDNAs**. The tree was created based on ORF sequences using the neighbor-joining method of the treepuzzle software. Branch lengths are measured relative to the estimated numbers of substitutions. Therefore, mGBP5 and mGBP5a appear less similar as they are actually on their amino acid level.

Using the maximum likelihood method we generated a phylogenetic tree of the mGBP family based on the ORF sequences (Fig. 5). This phylogenetic analysis revealed three predominant homology clusters. Two homology clusters are located on chromosome 3. The first homology cluster consists of *mGbp1*, *mGbp2*, and *mGbp5/5a*. The second homology cluster contains *mGbp3 *and *mGbp7*. The homologies of the members of these two clusters are lower than 60% (Table 2). The second cluster is with approximately 70% amino acid identities more closely related to the mGBPs in the third homology cluster on chromosome 5 encompassing *mGbp4*, *mGbp6, mGbp8*, *mGbp9*, *mGbp10*, and *mGbp11*. Within the cluster on chromosome 5 sequence identities reach 65% up to 98% (Table 2). The short branches implicate a just recent duplication of these genes on chromosome 5.

**Table 2:** Percent identities of the mGBPs based on ORF sequences. For the calculation of percent identities a multiple sequence alignment with the ClustalW algorithm using MegAlign (DNAStar) was performed. Percent identities greater than 80% are shown in bold.

**Percent Identities**												
	**mGBP1**	**mGBP2**	**mGBP3**	**mGBP4.1**	**mGBP5**	**mGBP5.a**	**mGBP6**	**mGBP7**	**mGBP8**	**mGBP9**	**mGBP10**	**mGBP11**

**mGBP1**	------	**82.4**	56.8	57.6	64.1	46.4	58.6	58.2	51.0	57.7	58.5	46.9
**mGBP2**		------	58.9	58.5	64.9	45.8	59.3	58.7	51.9	58.7	59.0	46.7
**mGBP3**			------	69.6	52.8	40.0	69.8	78.2	61.0	69.7	69.8	53.8
**mGBP4.1**				------	55.3	40.4	**86.9**	69.4	76.8	**87.0**	**86.8**	66.1
**mGBP5**					------	71.2	55.4	55.6	47.9	54.5	54.9	45.6
**mGBP5.a**						------	39.3	40.5	32.3	39.5	39.0	29.3
**mGBP6**							------	68.9	78.1	**90.0**	**98.4**	67.6
**mGBP7**								------	60.9	68.8	68.6	52.3
**mGBP8**									------	**90.2**	**91.3**	65.8
**mGBP9**										------	**88.7**	66.5
**mGBP10**											------	67.3
**mGBP11**												------

## Discussion

In order to find new IFNγ regulated host effector molecules we were able to identify and characterize five novel members of the mGBP family [5]. Further, we showed that all hitherto identified members of the mGBP family are IFNγ induced and moreover are highly upregulated in mice after infection with *L. monocytogene*s or *T. gondii*. Furthermore, we demonstrated that in infected cells most mGBPs surround the parasitophorous vacuole of *T. gondii *[5]. Consecutively, within this study, comprehensive homology and motif searches against public databases (NCBI, Ensembl) using EST and BAC sequences resolved the genomic organization and localization of the mGBPs in more detail. During these analyses we were able to identify the additional mGBP member mGBP11. The scope of this study was to determine the precise loci of the 11 mGBPs and of the two pseudogenes, to compare the structure and organization of the mGBPs, to compile all cDNA sequences, and to verify the expression of mGBP mRNAs.

### Genomic organization of the mGBPs

The combined analyses revealed two mGBP homology clusters on chromosomes 3 and 5. One mGBP cluster is located within the H3 region on chromosome 3 which is in contrast to previously published data where the cluster was mapped to the H1 region [21]. The second cluster is located in the E5 region on chromosome 5 which is in accordance to Olszewski et al. [21]. Furthermore, Olszewski et al. noted that within the mGBP cluster on chromosome 5 the only functional mGBP gene is *mGbp4 *and that in addition three pseudogenes (pseudo*mGbp2*, pseudo*mGbp3*, and pseudo*mGbp4*) are located on this chromosome. Our *in silico *and mRNA sequence analyses now clearly demonstrate that besides *mGbp4 *five expressed mGBPs (*mGbp6*, *mGbp8, mGbp9, mGbp10, and mGbp11*) are located on chromosome 5 (this study and [5]). In a previous report, we have shown that the *mGbp4 *locus does not encode for a complete mGBP4 protein [26]. For pseudo*mGbp2 *we could not find any corresponding EST sequence and were not able to clone a cDNA corresponding to pseudomGBP2, thus excluding the possibility of an alternative start downstream of exon 2. Based on BAC analyses we could demonstrate that the two pseudogenes pseudo*mGbp3 *and pseudo*mGbp4 *described by Olszewski et al. are both part of *mGbp8 *which was virtually disrupted by the *Abcg3 *gene locus in a former incorrect assembly within the public databases (see also [21]). Further transcript analyses revealed a functional *mGbp8 *locus and showed an IFNγ dependent upregulation of the mGBP8 mRNA comparable to mGBP6, mGBP9, mGBP10, and mGBP11. Similarly, all these mGBPs were highly induced upon *L. monocytogenes *infection in C57BL/6 mice (this study and [5]). The BAC sequences as well as our sequenced cDNA clones of the newly identified mGBP11 contain a premature stop codon within exon 8, leading to an ORF sequence of only 1329 bp. However, in the database (NCBI) one cDNA of mGBP11 without a premature stop was found (Acc. No. BC111039). It might be possible that the presence of different mGBP11 cDNAs is due to allelic variation. Further studies have to clarify, whether from this locus a functional protein can be translated and whether other mouse backgrounds differ in exon 8.

In a recently published report, some subtle differences to our extensive genomic analyses have been described [28]. Firstly, in this report no mGBP7 gene is presented. Secondly, mGBP6 in [28] is termed mGBP7 based on our NCBI database submission in 2006 (BK005760, [5]). Thirdly, mGBP12 has been deposited in 2007 as mGBP11 by us (EU304258, this study). To keep consistency between database and nomenclature we propose to refer to the mGBP assignment in Figure 1. This is also in accordance with the extensive protein sequence and functional analyses which were provided previously [5]. mGBP13 has been described as a pseudogene by Olszewski and here (see Fig. 1 pseudoGbp1). Unfortunately, no description of the methods used for the identification of the mGBP13 locus is given in [28]. However, further studies are required to confirm whether this is a functional mGBP locus.

Besides the chromosomal localization of the mGBPs we also elucidated the exon-intron structure of these genes. Interestingly, all mGBPs consist of eleven exons with an impressively similar gene organization, with only one exception in *mGbp8 *which lacks exon 6. In addition, the translation of all mGBPs starts in exon 2. For *mGbp3 *and *mGbp4 *alternative non-coding exons were found in the 5' regions. The usage of different 5' exons may influence the stability of the mRNAs [29]. Indeed, the frequencies of ESTs of mGBP3 and mGBP4 with the different alternative 5' exons are quite variable, so their functional significance has to be validated. It has been reported that mRNAs of genes with alternative 5' coding or non-coding exons are often expressed in a tissue-specific manner [30]. Further studies will be necessary to verify a potential tissue-specific expression/regulation of the different mRNA isoforms of mGBP3 and mGBP4.

### Evolution of the mGBPs

Recent data indicate that the GBPs are host effector molecules involved in pathogen defense [5,16,17]. Defense against pathogens requires permanent adaptation to the changes in pathogen virulence strategies [31,32]. We suggest that evolutionary pressure led to gene duplication events which have resulted in the current mGBP clusters on chromosomes 3 and 5. It is most likely, that these gene duplications started with one primordial mGBP. We suppose that this ancestor mGBP is located on chromosome 3 because these mGBPs are more divergent among each other as compared to the mGBPs on chromosome 5. Interestingly, on chromosome 3 two homology clusters have evolved. One homology cluster with *mGbp1*, *mGbp2*, and *mGbp5 *is characterized by a C-terminal CaaX motif for isoprenylation and a 'TLRD' G4 motif. In contrast, the second homology cluster with *mGbp3 *and *mGbp7 *lacks the CaaX motif and possess a 'TVRD' G4 motif. This finding leads to the hypothesis, that *mGbp3 *or *mGbp7 *is the ancestor for all mGBPs on chromosome 5 which also lack a CaaX motif and have a "TVRD" G4 motif. This is further corroborated by the high cDNA sequence identities (around 70%) of mGBP3 and mGBP7 with mGBP4, mGBP6, mGBP8, mGBP9, mGBP10, and mGBP11 on chromosome 5. On this chromosome also the most recent duplication event occurred, where *mGbp6 *emanated from *mGbp10 *or vice versa. This is supported by the high homology of 98.4% between these two GTPases. Moreover, we suggest that the mGBP cluster on chromosome 5 is a "genomic hot spot" permanently exposed to genetic recombination events. Consistent with this suggestion, we detected a transposon-like element (>1000 bp) which is integrated several times in this mGBP cluster (data not shown). Further studies have to clarify whether these genes evolved due to evolutionary pressure and whether these genes have redundant or non-redundant functions during pathogen defense.

We have shown that mGBPs are highly induced upon IFNγ stimulation and infection with intracellular bacteria or protozoa indicating an important role as effector molecules in host defense. Now, we describe and characterize the genomic loci of the mGBPs on chromosomes 3 and 5. These data will be very important for the analyses of evolutionary gene amplifications required in host defense as well as for functional studies by the generation of gene targeted mice which are under way.

## Methods

### Determination of the genomic sequences and localization of the mGBPs

To analyse the chromosomal localization of the mGBPs the mouse genome research page on the Ensembl website  was used. The genomic sequences of the appropriate chromosomal segments were determined using bacterial artificial chromosome (BAC) sequences. To display all available BACs on the contig view the corresponding field was activated using the 'decorations' function. For this study we have chosen the sequences of the BAC clones RP23-100J23 and RP24-314I8 for chromosome 3 and RP24-63G23, RP23-329M7, RP23-152O10, and RP24-210D14 for chromosome 5. We obtained the BACs' genomic sequences from the website of the National Center for Biotechnology Information (NCBI) . The sequences were downloaded [Accession numbers: AC102108 (RP23-100J23), AC115865 (RP24-314I8), AC113980 (RP24-63G23), AC123697 (RP23-329M7), AC144914 (RP24-210D14), and AC162798 (RP23-152O10)] and imported into EditSeq (DNAStar, Madison, WI) for assembly into contigs using the SeqMan II program (DNAStar, Madison, WI). For further analyses expressed sequence tags (ESTs) of mGBPs were obtained with the Basic Local Alignment Search Tool (BLAST) 'blastn' [25] from the NCBI website and mapped onto the respective genomic area.

### Identification of the exon-intron structure

The transcript sequences from mGBP4 (mpa-2) and mGBP6 (mpa-2l) were downloaded from the Ensembl website and imported into EditSeq. We then determined the exon-intron structure by using BLAST "align two sequences" using standard parameters on the NCBI website. The sequences of single exons from mGBP4 and mGBP6 were aligned with the corresponding BAC sequences. If the exons mapped to multiple regions on the BAC with high homology the regions were retained and imported into EditSeq. The equivalent sequences from the resulting BLAST hits on the BACs were subsequently aligned with MeqAlign (DNAStar, Madison, WI) using standard parameters. The 3' and 5' splice sites were identified manually by inspecting the alignment and confirmed by comparing the genomic sequences to the corresponding exon sequences. Once all exons were mapped on the genome the exons indicating a new gene locus were assembled and potential cDNAs were created. Finally, we determined the open reading frames by using the ORF search tool from EditSeq.

### Alignment and phylogenetic tree

The alignment of mGBP cDNAs was created with the software ClustalW [33] and the subsequent layout was done with JalView . The phylogenetic analysis was accomplished using the maximum likelihood method and treepuzzle  for construction of the phylogenetic tree. The treepuzzle software was run with the option for exact parameter estimates using the neighbor joining method. Finally, we used the software drawtree from the phylip package  to plot the tree data. All software was run on a Linux PC workstation.

### Cell culture and stimulation

The macrophage cell line ANA-1 [34] was cultured in very low endotoxin RPMI (Biochrom, Berlin, Germany) supplemented with 10% heat inactivated, low endotoxin fetal calf serum (Cambrex, Veniers, Belgium) and 50μM 2-β-Mercaptoethanol (Invitrogen, Karlsruhe, Germany).

For stimulation of ANA-1 cells we used 100 U/ml recombinant mouse IFNγ (R&D Systems, Mainz, Germany). After 16 h of IFNγ stimulation the cells were harvested for RNA preparation.

### Infection with Listeria monocytogenes

C57BL/6N mice were purchased from Charles River (Sulzfeld, Germany) and maintained in the animal facility of the Medical Faculty of the Heinrich-Heine-University under SPF conditions. All procedures performed on animals in this study have been approved by the Animal Care and Use Committee of the local government of Duesseldorf and have been in accordance with the German animal laws. C57BL/6N mice were intraperitoneally infected with 0.1 × LD_50 _*L. monocytogenes *(American type culture collection strain 43251), and organs were removed 48 h after infection.

### Amplification and cloning of mGBPs

Total RNA from cells and tissues was isolated using Trizol Reagent (Invitrogen) according to the manufacture's instructions. First-strand cDNA synthesis was performed using 1 μg of total RNA with M-MLV reverse transcriptase and oligo dT primer (Invitrogen). The subsequent PCR reactions were accomplished using specific forward and reverse primers (additional file 3), and sequencing for both DNA strands was done by GATC Biotech AG (Konstanz, Germany).

### Accession numbers of EST and cDNA sequences

NCBI accession numbers of EST and cDNA sequences for all mGBPs are listed. The cDNA sequence numbers are shown in bold. In the case of mGBP6 and mGBP10 some ESTs could not be assigned to one individual GBP due to sequence identities and are therefore grouped together.

#### mGBP-1

AW476703, BB033108, BI853322, BQ126171, BY575536, CA894196, CJ141369, **EF494422, NM_010259**

#### mGBP-2

AV337765, BB840634, BI249610, BI790719, BM933217, BM935622, BQ550593, BQ550594, CB574409, CF583081, CF583082, CJ055311, CJ056573, CJ057752, **NM_010260**

#### mGBP3

AA162247, AA170007, AA276469, AA276918, AA289618, AA289780, AA839370, AW228655, BB862339, BE282356, BE847132, BG861498, BG861928, BG862944, BG864332, BG865051, BG914515, BG915574, BG973623, BI105618, BI653801, BI653963, BI654072, BI654363, BI656878, BI656930, BI567265, BI658478, BI661510, BM120304, BM206838, BM210659, BM221385, BM222892, BM240875, BM244805, BP760734, BQ552945, BQ552946, BX528767, BY215748, BY329622, BY483332, BY487480, BY562117, CA541279, CA544763, CA546244, CA573999, CA577912, CF910901, CK329532, CK331660, CK389037, CX205022, **NM_018734, U44731, BC019195**

#### mGBP4

BB859245, BI661547, BY225616, CJ141442, CJ141546, **EF494424**, **NM_008620**

#### mGBP4.1

BF138720, BI659458, BY749043, **EF494423**

#### mGBP4+mGBP4.1

AA170248, AA866719, AW228052, BE227153, BE336008, BI659458, BG864747, BG865054, BM244843, BM244986, CF911085

#### mGBP5

AI021670, AV329198, BB022173, BB617729, BB634786, BF163382, BG863163, BI558563, BI662495, BY212549, BY214522, BY220452, BY221363, BY224819, **NM_153564**

#### mGBP6

AK128993, BC057969, **BC115768**, BF015742, BF015763, BK005759

#### mGBP10

BI853721, BG915468, BI853566, **DQ985743**

#### mGBP6 + mGBP10

AA140542, AA267762, BE687038, BG915105, BG916153, BG916251, BM239887, BM241485, BM242358, BM243209, BP764987, BX525828, BY765347, CA572886, CD351560, CJ062488, CK331259, CN660654

#### mGBP7

AA200741, AI226719, AW106727, BB554853, BB666410, BF452604, BG914537, BI657558, BK005760, BP766023, BX513589, BX522404, BY016011, BY178861, BY197520, BY215691, BY221698, BY221980, BY223566BY742706, BY 761161, BY761419, BY765425, BY765812, CA574325, CA576100, CA885708, CF899303, CJ137202, CJ140108, CK342680, CK342922, CK343278, CX207094, CX219108, DV057094

#### mGBP8

AA155498, BE686748, BM245207, BM245316, BM246467, BQ552741, **DQ295175**, BY717902, BI657260, CJ141546, CJ141512, **NM_029509**

#### mGBP9

AA122564, BE650518, BE692183, BE849117, BG915970, BY747136, CA535632, CJ165022, CJ183419, CJ164864, BY060909, BY064961, **DQ985742**, **NM_172777**

#### mGBP11

AA087907, **BC111039**, BI145047, BI655333, BF470780, BI660636

## Availability and requirements

To analyse the chromosomal localization of the mGBPs the mouse genome research page on the Ensembl website  was used. We obtained the BACs' genomic sequences from the website of the National Center for Biotechnology Information (NCBI) . The alignment of mGBP cDNAs was created with the software ClustalW [33] and the subsequent layout was done with JalView . The phylogenetic analysis was accomplished using the maximum likelihood method and treepuzzle  for construction of the phylogenetic tree. The treepuzzle software was run with the option for exact parameter estimates using the neighbor joining method. Finally, we used the software drawtree from the phylip package  to plot the tree data. All software was run on a Linux PC workstation.

## Authors' contributions

AK carried out the *in silico *analysis and drafted the manuscript. CK performed the PCRs and drafted the manuscript. JW participated in the sequence alignment and generated the phylogenetic tree. CBG carried out the infection experiments and stimulation of macrophages and prepared all cDNA. DD participated in the sequence alignment and drafted the manuscript. SB participated in the design of the study and drafted the manuscript. KP conceived the study, and participated in its design and coordination. All authors read and approved the final manuscript.

## Supplementary Material

Additional file 1 **ClustalW alignment of predicted protein sequences of the eleven 65 kDa mGBP members**. The alignment-layout was edited with the JalView software. Amino acids identical in all eleven mGBPs are marked in dark blue, identical in ≥75% in medium blue and identical in ≥50% in light blue. Red boxes highlight the conserved GTP binding motifs: P-loop (G1), Switch I (G2), Switch II (G3) and G4. The green box highlights the conserved CaaX motif. Click here for file

Additional file 2 **ClustalW alignment of ORF sequences of the eleven 65 kDa mGBP members**. The alignment-layout was edited with the JalView software. Basepairs identical in all eleven mGBPs are marked in dark blue, identical in ≥75% in medium blue and identical in ≥50% in light blue. Red boxes highlight the conserved GTP binding motifs: P-loop (G1), Switch I (G2), Switch II (G3) and G4. Click here for file

Additional file 3 **Primer pairs used for amplification of mGBP6, mGBP7, mGBP8, mGBP9, mGBP10, and mGBP11**. Due to the high homology an identical forward primer for mGBP8, mGBP9 and mGBP11 combined with specific reverse primers were used. For mGBP6 and mGBP10 an identical reverse primer and specific forward primers which bind in the 5' UTR were used. GAPDH served as an internal control. Click here for file
