# Supplementary material for: Analyses of murine GBP homology clusters based on in silico, in vitro and in vivo studies
Source: BMC Genomics. 2008 Apr 10;9:158. doi: 10.1186/1471-2164-9-158 (PMC2387175; doi:10.1186/1471-2164-9-158)
Supplement: Additional file 3 — Primer pairs used for amplification of mGBP6, mGBP7, mGBP8, mGBP9, mGBP10, and mGBP11. Due to the high homology an identical forward primer for mGBP8, mGBP9 and mGBP11 combined with specific reverse primers were used. For mGBP6 and mGBP10 an identical reverse primer and specific forward primers which bind in the 5' UTR were used. GAPDH served as an internal control. [file 1471-2164-9-158-S3.doc]

**Additional File 3**

|  | forward primer | reverse primer |
| --- | --- | --- |
| **mGBP6** | 5’-AGG GAG CCT GAG GAG GCA GC-3’ | 5’-GCG ACC GGT CCT AAA TGC TTT AGA TAA CGG AC-3’ |
| **mGBP7** | 5’-ATG GCA TCT GGT CCC A-3’ | 5’-CCT AGG TTT GGG AGT TTT CTA ACT TTG TCT G-3’ |
| **mGBP8** | 5’-ATG ACC CAA CCA CAA ATG G-3’ | 5’-ATC ATT GAA ATG GGA GAA-3’ |
| **mGBP9** | 5’-ATG ACC CAA CCA CAA ATG G-3’ | 5’-TCA TTT CCT TAA AAG TGA AGG ATT GT-3’ |
| **mGBP10** | 5’-ggt gat tcc tag gag aga gA-3’ | 5’-GCG ACC GGT CCT AAA TGC TTT AGA TAA CGG AC-3’ |
| **mGBP11** | 5’-ATG ACC CAA CCA CAA ATG G-3’ | 5’-TCA TTT GCT CCT AAA GAA TGA AA-3’ |
| **GAPDH** | 5’-CAT GTA GGC CAT GAG GTC CAC CAC-3’ | 5’-TGA AGG TCG GTG TGA ACG GAT TTG GC-3’ |
